# Exploring the impact of early life factors on inequalities in risk of overweight in UK children: findings from the UK Millennium Cohort Study

**DOI:** 10.1136/archdischild-2015-309465

**Published:** 2016-05-09

**Authors:** Samuel Massion, Sophie Wickham, Anna Pearce, Ben Barr, Catherine Law, David Taylor-Robinson

**Affiliations:** 1 Department of Public Health and Policy, University of Liverpool, Liverpool, UK; 2 Institute of Child Health, University College London, London, UK

**Keywords:** Child health, Obesity, Health inequalities, Socioeconomic circumstances, Smoking

## Abstract

**Background:**

Overweight and obesity in childhood are socially patterned, with higher prevalence in more disadvantaged populations, but it is unclear to what extent early life factors attenuate the social inequalities found in childhood overweight/obesity.

**Methods:**

We estimated relative risks (RRs) for being overweight (combining with obesity) at age 11 in 11 764 children from the UK Millennium Cohort Study (MCS) according to socio-economic circumstances (SEC). Early life risk factors were explored to assess if they attenuated associations between SECs and overweight.

**Results:**

28.84% of children were overweight at 11 years. Children of mothers with no academic qualifications were more likely to be overweight (RR 1.72, 95% CI 1.48 to 2.01) compared to children of mothers with degrees and higher degrees. Controlling for prenatal, perinatal, and early life characteristics (particularly maternal pre-pregnancy overweight and maternal smoking during pregnancy) reduced the RR for overweight to 1.44, 95% CI 1.23 to 1.69 in the group with the lowest academic qualifications compared to the highest.

**Conclusions:**

We observed a clear social gradient in overweight 11-year-old children using a representative UK sample. Moreover, we identified specific early life risk factors, including maternal smoking during pregnancy and maternal pre-pregnancy overweight, that partially account for the social inequalities found in childhood overweight. Policies to support mothers to maintain a healthy weight, breastfeed and abstain from smoking during pregnancy are important to improve maternal and child health outcomes, and our study provides some evidence that they may also help to address the continuing rise in inequalities in childhood overweight.

**What is already known on this topic**

- Childhood overweight and obesity is more common in disadvantaged children, but it is unclear the extent to which early life factors attenuate this relationship.

**What this study adds**

- In this large, nationally representative longitudinal study, inequality in overweight and obesity in preadolescence was partially attenuated by early life risk factors including maternal smoking during pregnancy and having a mother who was overweight before pregnancy.

## Introduction

### Background

Addressing the obesity epidemic is a global public health priority. In England, roughly one third of children aged between 2 and 15 are overweight/obese.^1^ Being overweight/obese increases the risk of developing type 2 diabetes, heart disease and some cancers.^2^ ^3^ Furthermore, childhood overweight/obesity is associated with social and psychological effects with increased risk of mental health problems, stigmatisation, social exclusion, low self-esteem, depression, and substance abuse.^3^ The cost of obesity to the has been estimated at over 5 billion per year in 2007 and is predicted to reach 50 billion per year in 2050.^4^

There are large social inequalities found in childhood overweight/obesity:^5^ A systematic review of 45 studies from a diverse pool of western developed countries found a consistent relationship between lower socio-economic circumstances (SEC) and obesity risk. The relationship was particularly strong using measures of maternal education as the SEC indicator. Compared to income and occupation, education is suggested to have a stronger influence on parenting behaviours in the pathway from low SEC to development of adiposity.^5^

Despite recent evidence suggesting a stabilisation of overweight/obesity prevalence in England,^6^ ^7^ socioeconomic inequalities in childhood overweight/obesity continue to widen.^7^ A number of studies suggest that early life risk factors, such as parental and maternal smoking during pregnancy, are predictive of childhood overweight/obesity.^8–11^ However, few studies have explored the extent to which these factors attenuate inequalities in overweight/obesity in later childhood. This study therefore aimed to assess whether early-life risk factors attenuate inequalities in overweight/obesity in 11-year-old children from the UK.

## Methods

### Design, setting, and data source

We used data from the Millennium Cohort Study (MCS), a nationally representative sample of children born in the UK between September 2000 and January 2002. Data were downloaded from the UK Data Archive in 2014. The study over-sampled children living in disadvantaged areas and those with high proportions of ethnic minority groups by means of stratified cluster sampling design.^12^ Further information on the cohort and sampling design can be found in the cohort profile.^12^ This study uses data collected on children at 9 months and 11 years. The analysis did not require additional ethical approval.

### Outcome measure: overweight (including obesity)

At 11 years trained investigators collected data on the height of children to the nearest 0.1 cm and weight to the nearest 0.1 kg. BMI was calculated by dividing weight (in kilos) by height squared (in metres). Being overweight (by combining overweight and obesity scores) was defined using the age and sex specific International Obesity Task Force (IOTF) cut-offs (baseline: thin or healthy weight).

### Exposure: SEC

The primary exposure of interest was maternal academic qualifications used as a fixed measure of SEC at birth of the MCS child. The highest qualification attained by the mother was established by questionnaire at the first wave, categorised in this study by six levels: degree plus (higher degree and first degree qualifications), diploma (in higher education), A-levels, grades A–C, GCSE grades D–G, and none of these qualifications.

### Mediators: early life risk factors

We examined the following early life risk factors associated with childhood overweight risk based on findings from a systematic review: (see ref. 13) *perinatal factors and exposures during pregnancy*: maternal pre-pregnancy overweight (yes or no); maternal smoking during pregnancy (none, 1–10 cigarettes per day (cpd), 11–20 cpd, >20 cpd); birthweight (normal, low, or high), preterm birth (yes or no), caesarean section (yes or no); *early life postnatal exposures* measured at 9 months: breastfeeding duration (never, less than 4 months, greater than 4 months), early introduction of solid foods (coded as <4 months yes/no as per Department of Health guidance at the time of the survey), and parity.

### Baseline confounding factors

Sex and ethnicity of child, and maternal age at birth of MCS child are associated with both exposure and outcome measures and so were considered as confounding factors.

### Analysis strategy

Following the Baron and Kenny steps to mediation,^14^ we explored the unadjusted association between maternal qualifications (primary exposure) and childhood overweight at 11 years (outcome measure). All analyses were conducted in STATA/SE V.13. We explored the associations between potential mediators and overweight, calculating unadjusted relative risks (RRs) using Poisson regression. Following this we explored the association between maternal qualifications and all potential mediators. In the final analysis sequential models were fitted; calculating adjusted RRs using Poisson regression for overweight on the basis of maternal qualification (with children of mothers with highest qualifications as the reference group), adjusting for the potential mediators that were significantly associated with overweight at the p<0.1 level in the univariate analysis.

We used a sequential approach to construct the adjusted models, first adding confounding variables, then perinatal factors and exposures during pregnancy, and finally postnatal exposures, to show the association between SEC and overweight. Mediation was taken to be a reduction in, or elimination of, statistically significant RRs in a final complete case sample.^15^ We estimated all model parameters using maximum likelihood, accounting for sample design and attrition. We undertook three sensitivity analyses, repeating the analysis with income as an alternative measure of SEC; calculating the relative index of inequality (RII); and also using the decomposition method.^16^ The results from the sensitivity analysis can be found in the online supplementary material.

10.1136/archdischild-2015-309465.supp1 Supplementary data

## Results

11 764 children were present at 9 months and 11 years with data on overweight status. 9424 (80%) had full data on all exposures of interest in the fully adjusted model. The prevalence of overweight at age 11 was 33.1% in children whose mother had lower qualifications, compared to 20.1% in the highest maternal qualification group (degree plus). All the other covariates of interest, except for sex, varied by level of maternal qualifications (table 1).

**Table 1:** Characteristics of the total study population, by level of maternal academic qualification at birth of child (n=11 764)

	Degree plus %	Diploma %	A levels %	GCSE A-C %	GCSE D–G %	None %	Total %	p Value*
Overweight/obese at age 11	20.1	26.8	26.3	29.7	33.2	33.1	28.7	<0.001
Child sex								
Male	52	54.9	50	52.6	51.7	51.8	52.2	0.56
Female	48	45.1	50	47.4	48.3	48.2	47.8	
Child ethnicity								
White	87.9	90.3	89.5	91.4	91.2	73.2	87.4	<0.001
Mixed	3.8	2.4	2.7	2.7	2.8	3.7	3.1	
Indian	2.3	1.2	1.9	0.9	0.7	2.8	1.6	
Pakistani	1	1	2.1	1.9	2.1	8.6	2.9	
Bangladeshi	0.2	0.6	0.8	0.6	0.7	3.7	1.1	
Black	3.3	4	2.2	1.9	1.8	5.4	2.9	
Other ethnic group	1.5	0.6	0.8	0.5	0.6	2.6	1.1	
Maternal age at MCS birth								
14–19	0.1	1.2	5.2	10.3	18.7	17.3	9.8	<0.001
20–24	2.8	10.7	16.2	20.5	28.7	27.7	18.8	
25–29	25.9	34	30.7	29.2	29.6	25.8	28.6	
30–34	43.1	35.4	30.7	26.5	16.7	18.7	27.6	
35+	28.1	18.7	17.2	13.5	6.3	10.5	15.1	
Maternal pre-pregnancy overweight								
No	78.1	70.1	71.1	68.4	67.2	69.3	70.5	<0.001
Yes	21.9	29.9	28.9	31.6	32.8	30.7	29.5	
Maternal smoking during pregnancy								
None	96.5	89	87.6	75	63.4	53.8	75.4	<0.001
1–10 cigs/day	3.2	9.1	10.3	20.3	28.7	30.4	18.6	
11–20 cigs/day	0.2	1.6	1.7	3.8	7.2	12.6	4.9	
>20 cigs/day	0.1	0.2	0.4	0.9	0.7	3.2	1.1	
Children in household								
1	49.8	45.4	43.9	40.3	45.7	31.8	41.6	<0.001
2 or 3	46.7	51.4	51.8	53.2	47.8	50.1	50.7	
4 or more	3.6	3.2	4.2	6.4	6.5	18.1	7.7	
Birthweight								
Normal	94.8	92.2	93.4	91.6	93	88.9	92.1	<0.001
Low	3.2	5.8	4.9	6.3	5.4	9.6	6.1	
High	1.9	2	1.7	2.1	1.6	1.5	1.9	
Preterm								
No	95.5	94.3	95.8	93.1	94.9	93.8	94.2	0.02
Yes	4.5	5.7	4.2	6.9	5.1	6.2	5.8	
Caesarean section								
No	75.1	76.7	79.9	80.6	80.2	83.9	79.9	<0.001
Yes	24.9	23.3	20.1	19.4	19.8	16.1	20.1	
Breastfeeding								
>4 months	56.2	33.8	35.7	19.3	11.2	13.1	26.2	<0.001
<4 months	36.9	46	45.1	43	37.5	33.4	40.1	
Never	6.9	20.2	19.2	37.7	51.2	53.5	33.7	
Solid foods before 4 months								
No	72.1	63.7	62.2	62.8	57.2	64.9	64	<0.001
Yes	27.9	36.3	37.8	37.2	42.8	35.1	36	

*χ^2^. All figures are percentages adjusted for sampling design. Missing data, unadjusted frequency: Sex 414, Ethnicity 1490, Maternal age 426, Maternal pre-pregnancy overweight 2072, Smoking 461, Children in household 414, Birthweight 1492, Preterm 1615, C-section 414, Breastfeeding 1481, Solid foods 424.

MCS, Millennium Cohort Study.

### Associations of covariates with overweight

**Figure 1:**
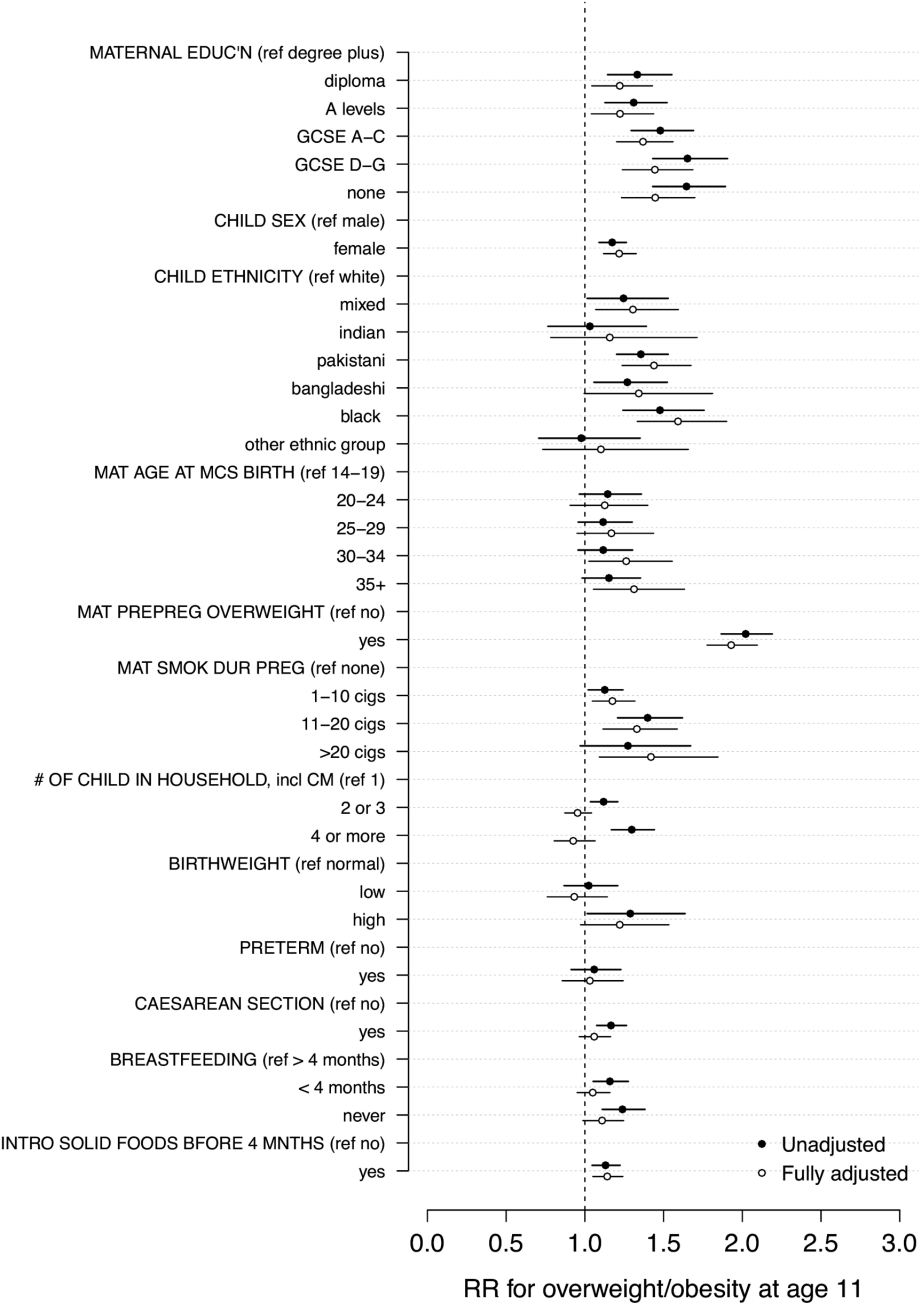
Univariate associations (relative risks, RRs) between covariates and overweight/obesity.

In the univariate regression, lower maternal qualifications, female sex, mixed, Pakistani, Bangladeshi and black ethnicity, maternal age of 35 and older at MCS birth, maternal pre-pregnancy overweight, maternal smoking during pregnancy, more than 1 child in the household, high birthweight, caesarean section, breastfeeding for less than 4 months, and introducing solid foods before 4 months were all associated with an increased RR for overweight in children at age 11 (table 2 and figure 1).

**Table 2:** Prevalence of overweight/obesity at age 11 and univariate RRs

	Total (N= 11764)	Overweight/Obese (N= 3445)	RR	Lower CI	Upper CI
	%	%			
Maternal education					
Degree plus	15.6	20.1	–	–	–
Diploma	8.2	26.8	1.33	1.14	1.55
A levels	9.3	26.3	1.31	1.13	1.52
GCSE A-C	35.8	29.7	1.48	1.29	1.69
GCSE D-G	12.1	33.2	1.65	1.43	1.91
None	19	33.1	1.65	1.43	1.89
Child sex					
Male	52.3	26.6	–	–	–
Female	47.7	31.2	1.17	1.09	1.26
Child ethnicity					
White	86.5	27.5	–	–	–
Mixed	3	34.2	1.25	1.01	1.53
Indian	1.8	28.4	1.03	0.77	1.39
Pakistani	3.2	37.3	1.36	1.20	1.53
Bangladeshi	1.2	34.9	1.27	1.06	1.52
Black	3.1	40.6	1.48	1.24	1.76
Other	1.2	26.9	0.98	0.71	1.35
Maternal age at MCS birth					
14−19	9.7	25.8	–	–	–
20−24	18.8	29.6	1.15	0.96	1.36
25−29	28.6	28.8	1.12	0.96	1.30
30−34	27.7	28.8	1.12	0.96	1.30
35+	15.2	29.8	1.15	0.98	1.35
Maternal pre-pregnancy overweight					
No	70.6	21.6	–	–	–
Yes	29.4	43.6	2.02	1.87	2.19
Smoking during pregnancy					
None	75.7	27.5	–	–	–
1−10 cigs/day	18.4	31	1.13	1.02	1.24
11−20 cigs/day	4.9	38.5	1.40	1.21	1.62
>20 cigs/day	1.1	35	1.27	0.97	1.67
Children in household					
1	41.8	26.5	–	–	–
2 or 3	50.5	29.7	1.12	1.04	1.21
4 or more	7.7	34.5	1.30	1.17	1.44
Birthweight					
Normal	92.1	28.3	–	–	–
Low	6	29	1.02	0.87	1.21
High	1.9	36.4	1.29	1.01	1.64
Preterm					
No	94.2	28.3	–	–	–
Yes	5.8	29.9	1.06	0.91	1.23
Cesarean section					
No	80	27.8	–	–	–
Yes	20	32.4	1.17	1.08	1.26
Breastfeeding					
>4 months	26.3	24.9	–	–	–
<4 months	40.1	28.8	1.16	1.05	1.28
Never	33.6	30.8	1.24	1.11	1.38
Solid food before 4 months					
No	64.5	27.5	–	–	–
Yes	35.5	31.1	1.13	1.05	1.22

MCS, Millennium Cohort Study; RR, relative risk.

Figure 1 shows the unadjusted and fully adjusted covariate estimates. In the fully adjusted model, lower maternal qualifications, female sex, mixed and Pakistani and Bangladeshi and black ethnicity, maternal age of 30 and older at MCS birth, maternal pre-pregnancy overweight, smoking during pregnancy, high birthweight, never breastfeeding, and introducing solid foods before 4 months were all significantly associated with an increase in RR for overweight. There was no significant effect associated with parity, low birthweight, having a preterm birth or caesarean section.

### Association between maternal academic qualifications and overweight, adjusted for other early life factors

**Figure 2:**
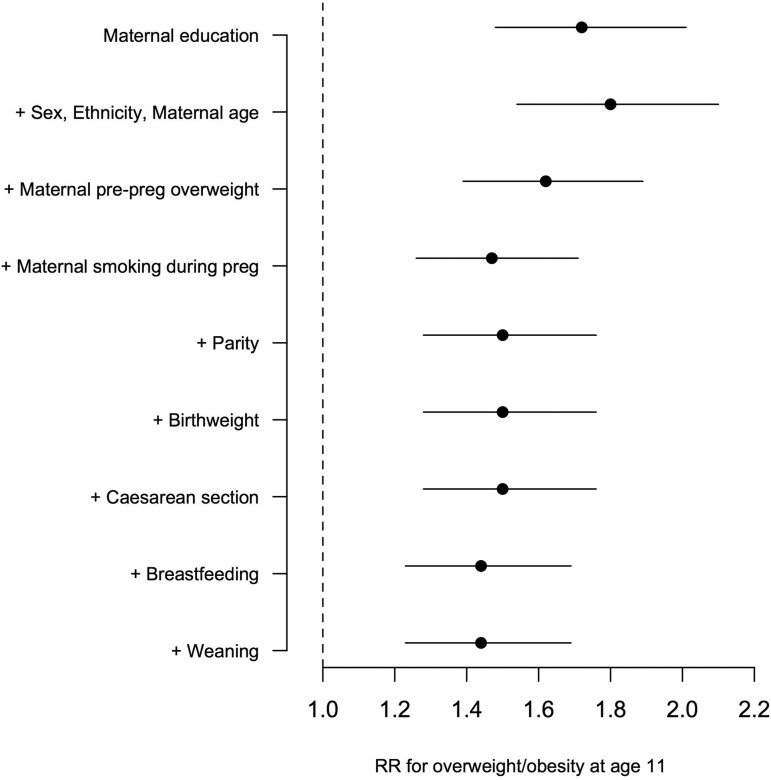
Relative Risk for obesity comparing lowest maternal academic qualification group to highest in sequentially adjusted model.

Figure 2 shows the RRs for maternal qualification and overweight before and after adjustment for covariates added sequentially using a life-course approach (see online supplementary material for data tables showing all the model coefficients). The RR increases from 1.72 (95% CI 1.48 to 2.01) to 1.80 (95% CI 1.54 to 2.10) after adjusting for confounders. There are incremental changes in the RR evident after adjusting for maternal pre-pregnancy overweight, maternal smoking during pregnancy, and breastfeeding. In the final full model, the RR comparing lowest to highest qualifications remains significant (1.44, 95% CI 1.23 to 1.69). Repeating the analysis, but only adding maternal pre-pregnancy overweight and maternal smoking during pregnancy to the confounder-adjusted model attenuated the RR to 1.47 (95% CI 1.26 to 1.71), indicating that the percentage of effect mediated by these factors equates to 41.3% (RR reduction).

### Sensitivity analysis

The conclusions of the study were similar when we used household income as the measure of SEC; when we used RII as the measure of inequality; and when we used an alternative method for mediation analysis (see online supplementary material).

## Discussion

Using a nationally representative sample, we show that overweight status at age 11 is socially patterned. Lower maternal qualifications, female sex, mixed and Pakistani and Bangladeshi and black ethnicity, maternal age of 30 and older at MCS birth, maternal pre-pregnancy overweight, smoking during pregnancy, high birthweight never breastfeeding, and introducing solid foods before 4 months were associated with an increase in RR for overweight at 11 years. Maternal pre-pregnancy overweight and maternal smoking during pregnancy attenuated the RR in the lowest maternal qualifications group by around 40% suggesting a considerable amount of the social inequalities in preadolescent overweight can be explained by these two variables.

### Comparison with other findings

Our study corroborates findings from a systematic review: Shrewsbury and Wardle^5^ found that 42% of the studies found an inverse association between SEC and adiposity, with the lowest SEC group having the highest level of adiposity. Using parental education as the SEC indicator, 75% of the studies demonstrated an inverse association between SEC and child adiposity. Children whose parents, particularly mothers, have lower levels of education are at a higher risk for developing adiposity. Shrewsbury and Wardle^5^ noted Sobal's theoretical framework that suggests education, as an indicator of SEC, influences knowledge and beliefs of parents, which is theorised to have more of an important role in the mechanism linking SEC and development of adiposity than other SEC indicators (eg, income and occupation). Though some of the studies in the review adjusted for confounding, none attempted to explore factors that attenuate the social gradient.

Our study is the first to quantify the contribution of early-life factors in attenuating social inequalities in overweight/obesity on the basis of maternal education level in a nationally representative sample of 11-year-old children in the UK. We found maternal pre-pregnancy overweight to be an important contributor to inequalities in overweight at 11 years, reducing the RR in the sequential model from 1.8 to 1.6. A recent study in a Dutch cohort found similar results, concluding that parental BMI, maternal pre-pregnancy BMI, and smoking during pregnancy contributed most to educational inequalities in BMI in 6-year-olds (attenuation −54%, 95% CI −98% to −33% in the lowest educational group).^9^ Maternal pre-pregnancy overweight is related to an increase risk of adverse health outcomes for mothers and infants including gestational diabetes, large baby size, and may produce other pre-programming effects related to increased risk in childhood overweight.^17^ ^18^ Parental overweight has also been found to potentially contribute to childhood overweight via family eating, activity, and factors in later life relating to child fat intake, snack consumption, and child preference for sedentary activities.^19–21^ These factors reflect the importance of addressing structural barriers to healthy eating faced by the parents of children growing up in more disadvantaged areas.

Our research findings are similar to a large Irish study investigating determinants of socioeconomic inequalities in obesity in Irish children which identified maternal smoking during pregnancy as a potential mediator.^11^ Potential mechanisms include impaired foetal growth followed by rapid infant weight gain;^10^ the influence of prenatal smoking on neural regulation causing increased appetite and decreased physical activity;^22^ the associations of smoking with other health damaging behaviours after birth;^23^ and the contribution of smoking to family poverty, leading to constrained food budgets and fuelling the consumption of cheap, poor quality foods.^11^ Further longitudinal research efforts should be dedicated towards discovering the underlying mechanisms linking prenatal smoking to childhood overweight, and the extent to which they may also explain inequalities.

Our study suggests that shorter duration of breastfeeding may make a small contribution to the increased risk of preadolescent overweight in more disadvantaged children. Never breastfeeding was associated with a significantly higher risk of overweight in children at 11 years in the fully adjusted model, corroborating a previous study on the MCS data at an earlier age.^8^

### Strengths and limitations

This study used secondary data from a large, contemporary UK cohort and the results are likely to be generalisable to other high-income countries. A wide range of information is collected in the MCS, which allowed us to explore a range of prenatal, perinatal, and early life risk factors for overweight, including different measures of SEC. Overweight status was based on IOTF cut-offs for BMI, age and sex specific. Children's BMI was calculated based on height and weight measures taken by trained interviewers, reducing reporting bias of family members. However, using BMI as an indicator for adiposity may not be as accurate as measuring total fat mass.^9^

Missing data are a ubiquitous problem in cohort studies. Sampling and response weights were used in all analyses here to account for the sampling design and attrition to age 11. A complete case analysis was used, removing individuals with incomplete data. This approach may introduce bias, when the individuals who are excluded are not a random sample from the target population. However in this analysis the sample was sufficiently large, and the internal associations, which were the targets of inference within the sample population, are likely to be valid, but we speculate that they may underestimate the effect sizes in the full UK population.

In our analysis of mediation we have followed the Baron and Kenny approach.^14^ We used multiple measures of SECs, all of which further supported our main findings. These alternative methods for mediation analysis are continuously being developed, and have their limitations.^24^ For example, the KHB model using logistic regression as Poisson regression results were considered “experimental”. In this respect our analysis is exploratory and opens up the possibility of more focused mediation analyses to quantify the mediating pathways for specific factors identified in our study.

The positive association between maternal and child overweight may in part reflect non-modifiable (eg, genetic) factors. Furthermore, maternal smoking in pregnancy may itself be a proxy marker for SEC. However, we did observe a dose-response relationship between smoking in pregnancy and overweight/obesity, and the association remained after adjusting for multiple measures of SECs, supporting the notion of a causal link.

Finally, in the absence of randomised control trial data to assess the causal relationship between early life risk factors and childhood overweight, we are reliant on the best quality evidence from prospective observational studies. The systematic review of observational studies by Weng *et al*,^13^ concludes there is “strong evidence that maternal pre-pregnancy overweight and maternal smoking in pregnancy increased the likelihood of childhood overweight”. In a nationally representative contemporary cohort of UK children we have shown that maternal overweight and smoking during pregnancy may also account for a significant proportion of the social inequality in overweight/obesity. However, as Weng *et al* point out, the association between smoking and obesity/overweight may be confounded by other lifestyle factors, such as poor diet.

### Policy and practice implications

Policies to support mothers to maintain a healthy weight, breastfeed and abstain from smoking during pregnancy are important to improve maternal and child health outcomes, and our study provides some evidence that they may also help to address the continuing rise in inequalities in childhood overweight. Policies should focus on supporting access to healthy diets, particularly in the pre-conception and antenatal periods, and making healthy eating affordable for disadvantaged families. Future research aimed at reducing childhood obesity should also assess the inequalities impact of interventions in order to build the evidence base to reduce the large social inequalities found in overweight/obesity in childhood.
