# Supplementary material for: Exploring the impact of early life factors on inequalities in risk of overweight in UK children: findings from the UK Millennium Cohort Study
Source: Arch Dis Child. 2016 May 9;101(8):724–30. doi: 10.1136/archdischild-2015-309465 (PMC4975808; doi:10.1136/archdischild-2015-309465)
Supplement: Supplementary data [file archdischild-2015-309465supp.pdf]

**Online only supplementary material**

# Exploring the impact of early life factors on inequalities in risk of overweight in UK children: Findings from the UK Millennium Cohort Study

Samuel Massion, Sophie Wickham, Anna Pearce, Ben Barr,
Catherine Law, David Taylor-Robinson

This data supplement contains additional information on the methods employed in the study. In addition, further plots and results are presented.

## Results

### Table E1: Relative risk ratios (RRR) for overweight/obesity (all coefficients)

1. Maternal education
2. Maternal education + sex + ethnicity + maternal age at birth of child
3. Maternal education + sex + ethnicity + maternal age at birth of child+ maternal prepreg overweight
4. Maternal education + sex + ethnicity + maternal age at birth of child+ maternal prepreg overweight + smoking during pregnancy
5. Maternal education + sex + ethnicity + maternal age at birth of child+ maternal prepreg overweight + smoking during pregnancy + parity
6. Maternal education + sex + ethnicity + maternal age at birth of child+ maternal prepreg overweight + smoking during pregnancy + parity + birthweight
7. Maternal education + sex + ethnicity + maternal age at birth of child+ maternal prepreg overweight + smoking during pregnancy + parity + birthweight + C-section
8. Maternal education + sex + ethnicity + maternal age at birth of child+ maternal prepreg overweight + smoking during pregnancy + parity + birthweight + C-section + breastfeeding
9. Maternal education + sex + ethnicity + maternal age at birth of child+ maternal prepreg overweight + smoking during pregnancy + parity + birthweight + C-section + breastfeeding + intro to solid foods

| | 1 | 2 | 3 | 4 | 5 | 6 | 7 | 8 | 9 |
|---|---|---|---|---|---|---|---|---|---|
| degree plus | 1.00 | 1.00 | 1.00 | 1.00 | 1.00 | 1.00 | 1.00 | 1.00 | 1.00 |
| diploma | 1.31 | 1.34 | 1.26 | 1.24 | 1.25 | 1.25 | 1.25 | 1.22 | 1.22 |
| min 95 | 1.11 | 1.14 | 1.07 | 1.06 | 1.06 | 1.07 | 1.06 | 1.05 | 1.04 |
| max 95 | 1.55 | 1.57 | 1.47 | 1.45 | 1.46 | 1.46 | 1.46 | 1.43 | 1.42 |
| A levels | 1.29 | 1.33 | 1.24 | 1.23 | 1.23 | 1.23 | 1.24 | 1.22 | 1.21 |
| min 95 | 1.09 | 1.13 | 1.06 | 1.05 | 1.05 | 1.05 | 1.05 | 1.04 | 1.03 |
| max 95 | 1.52 | 1.56 | 1.45 | 1.44 | 1.45 | 1.45 | 1.45 | 1.43 | 1.42 |
| GCSE A-C | 1.50 | 1.58 | 1.44 | 1.39 | 1.41 | 1.41 | 1.41 | 1.37 | 1.36 |
| min 95 | 1.31 | 1.39 | 1.27 | 1.23 | 1.24 | 1.24 | 1.24 | 1.20 | 1.19 |
| max 95 | 1.73 | 1.81 | 1.64 | 1.58 | 1.60 | 1.60 | 1.60 | 1.56 | 1.55 |
| GCSE D-G | 1.61 | 1.75 | 1.55 | 1.47 | 1.48 | 1.49 | 1.48 | 1.43 | 1.42 |
| min 95 | 1.38 | 1.49 | 1.33 | 1.26 | 1.27 | 1.28 | 1.27 | 1.22 | 1.21 |
| max 95 | 1.89 | 2.05 | 1.80 | 1.71 | 1.73 | 1.73 | 1.73 | 1.67 | 1.65 |
| none | 1.72 | 1.80 | 1.62 | 1.47 | 1.50 | 1.50 | 1.50 | 1.44 | 1.44 |
| min 95 | 1.48 | 1.54 | 1.39 | 1.26 | 1.28 | 1.28 | 1.28 | 1.23 | 1.23 |
| max 95 | 2.01 | 2.10 | 1.89 | 1.71 | 1.76 | 1.76 | 1.76 | 1.69 | 1.69 |
| male | | 1.00 | 1.00 | 1.00 | 1.00 | 1.00 | 1.00 | 1.00 | 1.00 |
| female | | 1.19 | 1.18 | 1.19 | 1.19 | 1.19 | 1.20 | 1.19 | 1.21 |
| min 95 | | 1.09 | 1.09 | 1.09 | 1.09 | 1.10 | 1.10 | 1.10 | 1.11 |
| max 95 | | 1.29 | 1.29 | 1.29 | 1.29 | 1.30 | 1.30 | 1.30 | 1.31 |
| white | | 1.00 | 1.00 | 1.00 | 1.00 | 1.00 | 1.00 | 1.00 | 1.00 |

| | | | | | | | | | |
|---|---|---|---|---|---|---|---|---|---|
| mixed | | 1.28 | 1.27 | 1.27 | 1.27 | 1.27 | 1.27 | 1.30 | 1.30 |
| min 95 | | 1.03 | 1.04 | 1.04 | 1.04 | 1.04 | 1.04 | 1.06 | 1.06 |
| max 95 | | 1.58 | 1.55 | 1.55 | 1.55 | 1.56 | 1.56 | 1.59 | 1.59 |
| indian | | 0.98 | 1.01 | 1.08 | 1.08 | 1.09 | 1.10 | 1.12 | 1.14 |
| min 95 | | 0.65 | 0.68 | 0.72 | 0.72 | 0.73 | 0.74 | 0.76 | 0.77 |
| max 95 | | 1.47 | 1.51 | 1.61 | 1.61 | 1.63 | 1.63 | 1.65 | 1.69 |
| pakistani | | 1.32 | 1.26 | 1.37 | 1.38 | 1.39 | 1.39 | 1.41 | 1.46 |
| min 95 | | 1.14 | 1.10 | 1.19 | 1.20 | 1.21 | 1.21 | 1.22 | 1.26 |
| max 95 | | 1.52 | 1.43 | 1.56 | 1.58 | 1.60 | 1.60 | 1.62 | 1.68 |
| bangladeshi | | 1.15 | 1.13 | 1.24 | 1.26 | 1.27 | 1.26 | 1.29 | 1.33 |
| min 95 | | 0.85 | 0.84 | 0.92 | 0.93 | 0.94 | 0.93 | 0.96 | 0.99 |
| max 95 | | 1.56 | 1.52 | 1.67 | 1.70 | 1.71 | 1.70 | 1.74 | 1.79 |
| black | | 1.60 | 1.45 | 1.49 | 1.50 | 1.51 | 1.50 | 1.55 | 1.57 |
| min 95 | | 1.33 | 1.22 | 1.26 | 1.27 | 1.27 | 1.27 | 1.30 | 1.32 |
| max 95 | | 1.93 | 1.72 | 1.77 | 1.78 | 1.78 | 1.78 | 1.84 | 1.88 |
| other ethnic group | | 1.00 | 1.02 | 1.09 | 1.10 | 1.11 | 1.10 | 1.14 | 1.17 |
| min 95 | | 0.64 | 0.68 | 0.73 | 0.73 | 0.74 | 0.73 | 0.76 | 0.79 |
| max 95 | | 1.56 | 1.54 | 1.65 | 1.65 | 1.66 | 1.65 | 1.71 | 1.73 |
| 14-19 (maternal age) | | 1.00 | 1.00 | 1.00 | 1.00 | 1.00 | 1.00 | 1.00 | 1.00 |
| 20-24 | | 1.27 | 1.10 | 1.10 | 1.12 | 1.12 | 1.12 | 1.13 | 1.13 |
| min 95 | | 1.04 | 0.90 | 0.90 | 0.91 | 0.91 | 0.90 | 0.91 | 0.91 |
| max 95 | | 1.55 | 1.34 | 1.35 | 1.38 | 1.38 | 1.38 | 1.39 | 1.39 |
| 25-29 | | 1.31 | 1.10 | 1.12 | 1.15 | 1.15 | 1.15 | 1.17 | 1.16 |
| min 95 | | 1.08 | 0.91 | 0.93 | 0.94 | 0.94 | 0.94 | 0.95 | 0.95 |
| max 95 | | 1.59 | 1.33 | 1.36 | 1.40 | 1.41 | 1.40 | 1.43 | 1.42 |
| 30-34 | | 1.37 | 1.15 | 1.18 | 1.22 | 1.22 | 1.21 | 1.24 | 1.25 |
| min 95 | | 1.14 | 0.96 | 0.98 | 0.99 | 1.00 | 0.99 | 1.01 | 1.02 |
| max 95 | | 1.65 | 1.38 | 1.42 | 1.49 | 1.50 | 1.49 | 1.53 | 1.53 |
| 35+ | | 1.41 | 1.17 | 1.21 | 1.25 | 1.26 | 1.24 | 1.28 | 1.29 |
| min 95 | | 1.16 | 0.96 | 0.99 | 1.01 | 1.02 | 1.00 | 1.03 | 1.04 |
| max 95 | | 1.71 | 1.42 | 1.47 | 1.54 | 1.55 | 1.54 | 1.59 | 1.61 |
| maternal pre-pregnancy normal | | | 1.00 | 1.00 | 1.00 | 1.00 | 1.00 | 1.00 | 1.00 |
| maternal pre-pregnancy overweight | | | 1.96 | 1.96 | 1.97 | 1.96 | 1.95 | 1.94 | 1.93 |
| min 95 | | | 1.81 | 1.81 | 1.82 | 1.81 | 1.79 | 1.79 | 1.78 |
| max 95 | | | 2.12 | 2.12 | 2.13 | 2.12 | 2.11 | 2.11 | 2.10 |
| never smoked during pregnancy | | | | 1.00 | 1.00 | 1.00 | 1.00 | 1.00 | 1.00 |
| 1-10 cigs/day | | | | 1.18 | 1.18 | 1.19 | 1.19 | 1.17 | 1.16 |
| min 95 | | | | 1.05 | 1.06 | 1.06 | 1.06 | 1.05 | 1.04 |
| max 95 | | | | 1.32 | 1.32 | 1.33 | 1.33 | 1.32 | 1.30 |
| 11-20 cigs/day | | | | 1.37 | 1.38 | 1.39 | 1.39 | 1.37 | 1.34 |

| | | | | | | | | | | |
|---|---|---|---|---|---|---|---|---|---|---|
| min 95 | | | | 1.15 | 1.16 | 1.16 | 1.16 | 1.15 | 1.12 |
| max 95 | | | | 1.63 | 1.65 | 1.65 | 1.65 | 1.63 | 1.60 |
| >20 cigs/day | | | | 1.35 | 1.37 | 1.38 | 1.38 | 1.36 | 1.35 |
| min 95 | | | | 1.03 | 1.05 | 1.06 | 1.06 | 1.04 | 1.02 |
| max 95 | | | | 1.77 | 1.79 | 1.80 | 1.80 | 1.78 | 1.77 |
| 1 child in household (incl CM) | | | | | 1.00 | 1.00 | 1.00 | 1.00 | 1.00 |
| 2 or 3 | | | | | 0.96 | 0.96 | 0.96 | 0.96 | 0.95 |
| min 95 | | | | | 0.88 | 0.88 | 0.88 | 0.88 | 0.87 |
| max 95 | | | | | 1.05 | 1.05 | 1.06 | 1.05 | 1.04 |
| 4 or more | | | | | 0.92 | 0.92 | 0.93 | 0.92 | 0.92 |
| min 95 | | | | | 0.81 | 0.80 | 0.81 | 0.80 | 0.80 |
| max 95 | | | | | 1.06 | 1.06 | 1.07 | 1.06 | 1.06 |
| normal birthweight | | | | | | 1.00 | 1.00 | 1.00 | 1.00 |
| low birthweight | | | | | | 0.96 | 0.94 | 0.94 | 0.95 |
| min 95 | | | | | | 0.81 | 0.80 | 0.80 | 0.81 |
| max 95 | | | | | | 1.13 | 1.11 | 1.11 | 1.13 |
| high birthweight | | | | | | 1.22 | 1.22 | 1.22 | 1.21 |
| min 95 | | | | | | 0.97 | 0.97 | 0.97 | 0.97 |
| max 95 | | | | | | 1.53 | 1.53 | 1.54 | 1.52 |
| No Caesarean section | | | | | | | 1.00 | 1.00 | 1.00 |
| Caesarean section | | | | | | | 1.06 | 1.05 | 1.05 |
| min 95 | | | | | | | 0.96 | 0.96 | 0.96 |
| max 95 | | | | | | | 1.16 | 1.16 | 1.16 |
| Breastfed for >4months | | | | | | | | 1.00 | 1.00 |
| <4 months | | | | | | | | 1.07 | 1.06 |
| min 95 | | | | | | | | 0.97 | 0.96 |
| max 95 | | | | | | | | 1.18 | 1.17 |
| never breastfed | | | | | | | | 1.13 | 1.12 |
| min 95 | | | | | | | | 1.01 | 0.99 |
| max 95 | | | | | | | | 1.27 | 1.26 |
| Did not introduce solid foods before 4 months | | | | | | | | | 1.00 |
| introduced solid foods before 4 months | | | | | | | | | 1.15 |
| min 95 | | | | | | | | | 1.05 |
| max 95 | | | | | | | | | 1.24 |

*Sensitivity Analyses*
*Income quintiles as a measure of SEC*

Using income quintile at 9 months as a measure of SEC, instead of maternal academic qualifications, showed similar patterns with increased RR for overweight as household income decreased. The RR comparing lowest income quintile compared to highest after adjustment for confounders was 1.67 (95% CI: 1.44 to 1.94). After adjusting for all the covariates of interest, the RR comparing lowest to highest income quintiles remains significant (1.42, 95% CI: 1.22 to 1.65). Maternal pre-pregnancy overweight and maternal smoking during pregnancy were the primary contributors to the attenuation in the RR after adjusting for the potential mediators.

**Figure E1. Relative risk ratio for overweight/obesity comparing highest income quintile to lowest in sequentially adjusted models.** The plot shows partial attenuation of increased relative risk in children of lowest income households compared to highest with sequential adjustment for early life factors.

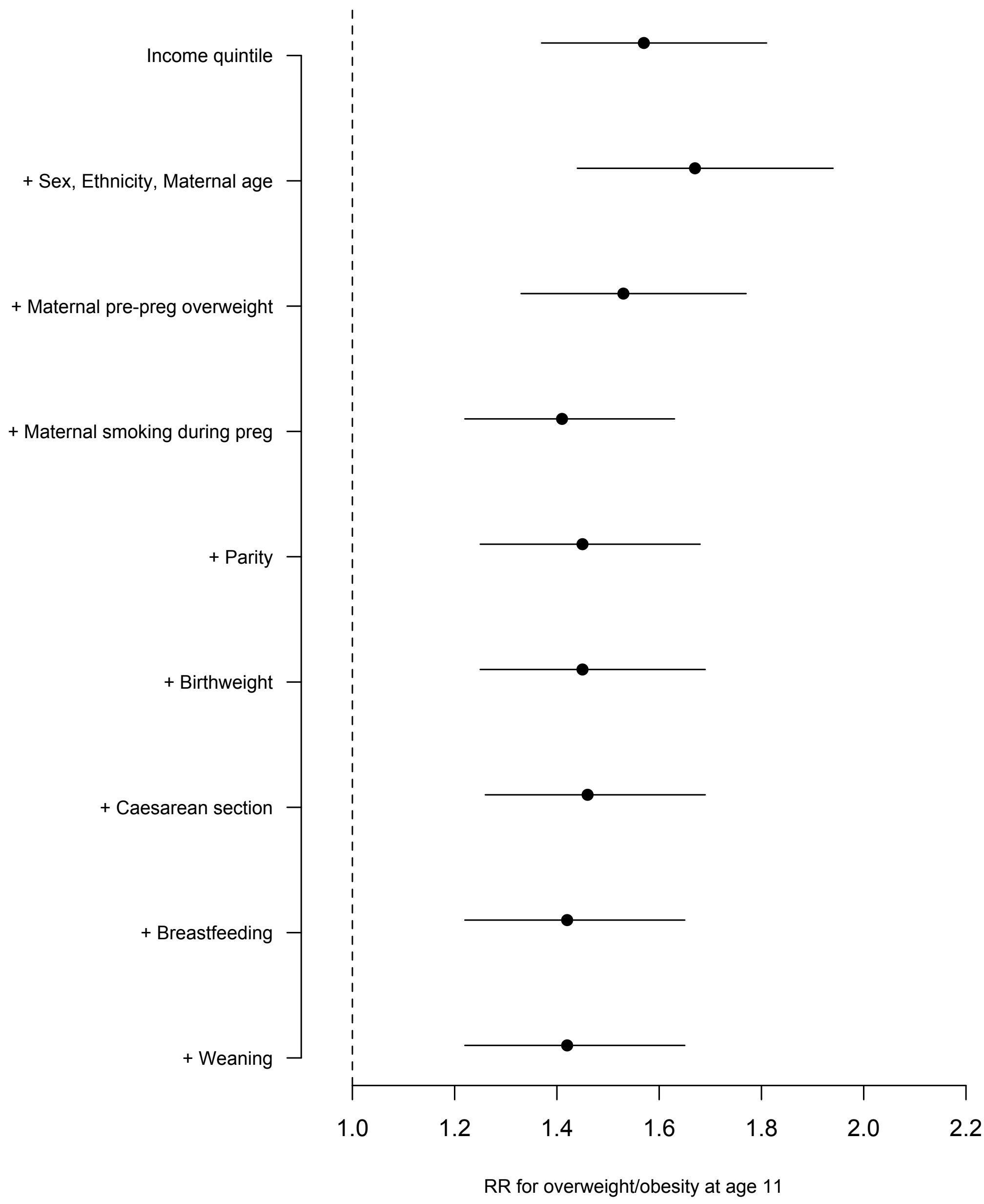

*Using the relative index of inequalities*

The plot shows how the social gradient in obesity/overweight changes after adjustment for covariates of interest. The blue line shows the gradient in the log odds of overweight/obesity by maternal education in the unadjusted model and the red line shows the gradient in the fully adjusted model (as seen in Table E1). The relative index of inequality (RII) can be derived from the slope of these lines, and interpreted as the relative risk ratios at the top of the social hierarchy compared to the bottom, taking into account the whole distribution of the population.[1]

**Figure E2. Calculating the relative index of inequality (RII) for overweight before and after adjustment for covariates.**

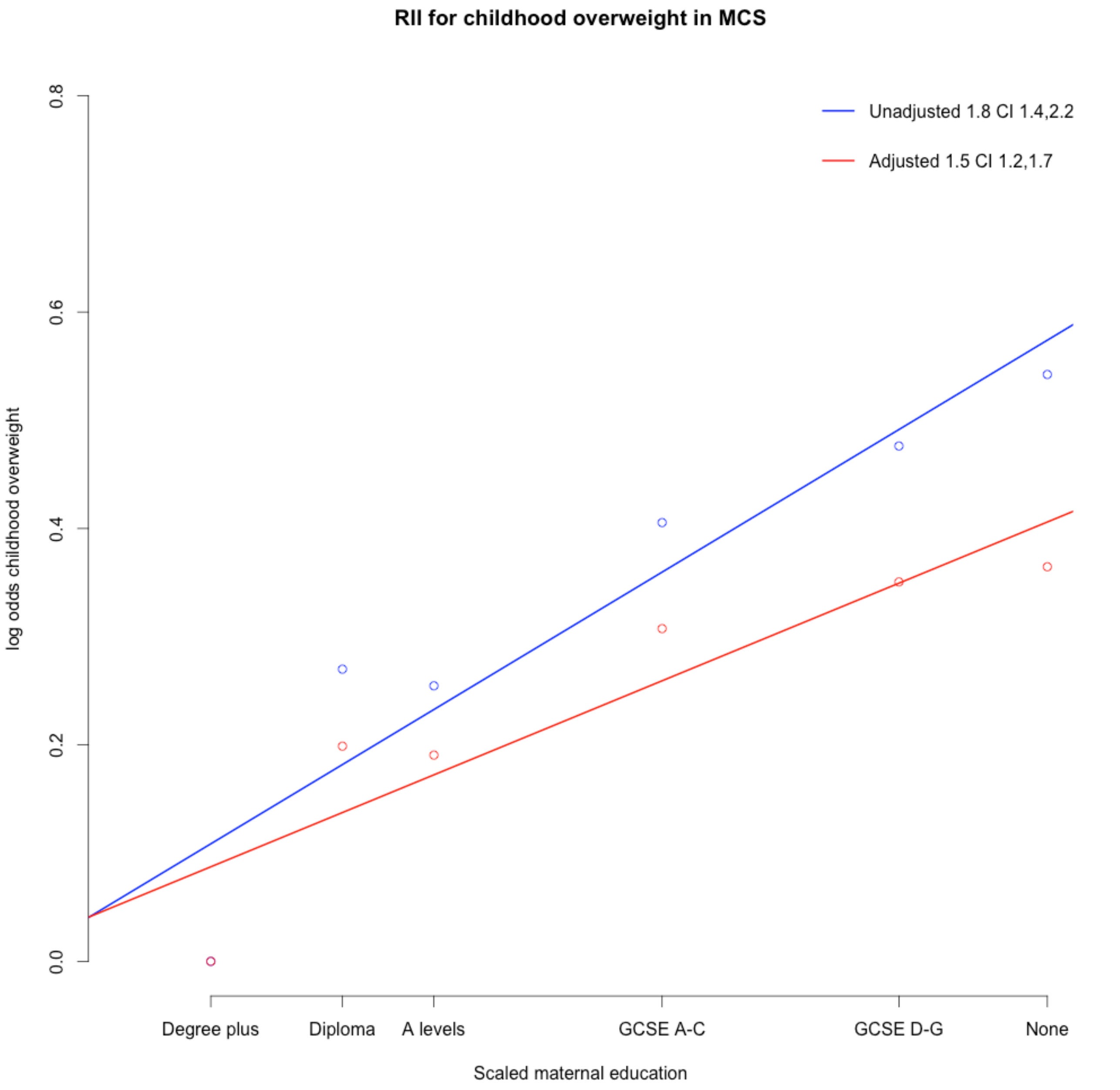

*Using the KHB method*

The number of mediators possible using this method is restricted; therefore, mediators were chosen based on the adjusted model described in the main analysis. The strength of this model is that it expresses the contribution of each mediator to the indirect effect to the outcome variable (inequalities in overweight). Using the KHB decomposition method,[2] an alternative form of mediation analysis, found that 34.4% of the total effect of having a mother with no academic qualifications on overweight in children was attributed to maternal smoking during pregnancy and maternal pre pregnancy overweight. This broadly supports the findings from our sequential approach model.

**Table E2: Decomposition using the KHB method, Summary of Confounding.** The confounding ratio tells us that the total, or unadjusted, effect of no maternal education on overweight is 1.52 times larger than the direct, or adjusted, effect. The confounding percentage tells us that 34.41% of the total effect of no maternal education is due to having a mother that is overweight before pregnancy and maternal smoking during pregnancy.

| | Confounding ratio | Confounding percentage | Rescale Factor |
|---|---|---|---|
| Degree plus | - | - | - |
| diploma | 1.34 | 25.41 | 1.01 |
| A-levels | 1.39 | 28.08 | 1.01 |
| GCSE A-C | 1.38 | 27.71 | 1.02 |
| GCSE D-G | 1.45 | 30.96 | 1.02 |
| none | 1.52 | 34.41 | 1.02 |

**Table E3: Decomposition using the KHB method, Components of Difference.** The first two columns of the disentangle table show the indirect effect (coefficient of reduced model – coefficient of full model) due to each of the mediators along with their standard errors. The values in the first column sum up to 0.26, the sum of indirect effects by all mediators. The third column expresses the contribution of each mediator to the indirect effect, and the last column shows how much of the total effect is due to confounding of the respective mediator; this last column sums up to 34.41, the overall confounding percentage. According to the results shown here, the degree of mediation is very slightly less for maternal pre-pregnancy overweight than for maternal smoking during pregnancy.

| | Coefficient | Standard Error | P_Diff | P_Red |
|---|---|---|---|---|
| **Degree plus** | | | | |
| maternal pre-pregnancy overweight | 0 | 0 | - | - |
| 1-10 cigs/day | 0 | 0 | - | - |

| | | | | |
|---|---|---|---|---|
| 11-20 cigs/day | 0 | 0 | - | - |
| >20 cigs/day | 0 | 0 | - | - |
| **None** | | | | |
| maternal pre-pregnancy overweight | 0.13 | 0.02 | 46.57 | 16.03 |
| 1-10 cigs/day | 0.06 | 0.02 | 20.73 | 7.13 |
| 11-20 cigs/day | 0.08 | 0.02 | 26.40 | 9.09 |
| >20 cigs/day | 0.02 | 0.01 | 6.30 | 2.17 |

## References

1 Hayes LJ, Berry G. Sampling variability of the Kunst-Mackenbach relative index of inequality. *Journal of Epidemiology and Community Health* 2002;**56**:762–5. doi:10.1136/jech.56.10.762

2 Kohler U, Karlson KB, Holm A. Comparing coefficients of nested nonlinear probability models. *The Stata Journal* 2011;**11**:420–38. doi:The Stata Journal
